# Developing a Risk Stratification Tool to Predict Patients with Gestational Diabetes Mellitus at Risk of Insulin Treatment: A Cohort Study

**DOI:** 10.3390/jpm15060223

**Published:** 2025-05-30

**Authors:** Xi Yang, Hannah L. Nathan, Ebruba E. Oyekan, Tim I. M. Korevaar, Doaa Ahmed, Katherine Pacifico, Aisha Hameed, Manju Chandiramani, Anita Banerjee, Caroline Ovadia

**Affiliations:** 1 Department of Women and Children’s Heath, King’s College London, London SE1 7EH, UK; xi.yang@imperial.ac.uk (X.Y.); manju.chandiramani1@gstt.nhs.uk (M.C.); anita.banerjee@gstt.nhs.uk (A.B.); 2 Institute of Reproductive and Developmental Biology, Imperial College London, London W12 0NN, UK; 3 Obstetrics and Gynaecology Division of Women’s Health, Princess Royal University Hospital, Kings College Hospital NHS Foundation Trust, London SE5 9RS, UK; hannah.nathan@nhs.net (H.L.N.); ebruba.ese@nhs.net (E.E.O.); aisha.hameed@nhs.net (A.H.); 4 Department of Internal Medicine and Academic Center for Thyroid Diseases, Erasmus University Medical Center, 3015 GD Rotterdam, The Netherlands; t.korevaar@erasmusmc.nl; 5 Women’s Services, Guys’ and St Thomas’ NHS Foundation Trust, London SE1 9RT, UK; doaa.ahmed@gstt.nhs.uk (D.A.);

**Keywords:** gestational diabetes, insulin requirement, risk stratification tool, predictive model

## Abstract

**Objectives:** We aimed to develop and validate a simple, easy-to-use risk stratification tool to use in the diagnosis of gestational diabetes mellitus (GDM) to triage those more likely to require insulin treatment. **Methods**: Using an audit of patients with GDM in 2019, multivariable logistic regression was used to select variables and develop a prediction model for insulin requirement. A stratification tool was developed by dichotomising these selected variables; its performance was assessed with an internal cohort from 2021 and externally from patients managed at a separate hospital. **Results**: Patients with a higher fasting blood glucose concentration (OR 2.41, 95% CI 1.84–3.15) and higher booking body mass index (OR 1.48, 95% CI 1.07–2.03) were more likely to require insulin therapy whilst a later gestational-weeks-at-diagnosis value gave a lower risk of insulin therapy (OR 0.71, 95% CI 0.62–0.81 per week). The low-risk group for insulin requirement was defined thus: fasting blood glucose < 5.6 mmol/L, booking BMI < 30 kg/m^2^, and gestational weeks at diagnosis ≥ 24 weeks. This classification had a negative predictive value (NPV) of 94% for insulin requirement, with a sensitivity of 84% and specificity of 56% in the development cohort. Similarly, in the internal and external validation cohorts, the NPVs were 93 and 90%, with sensitivity values of 77 and 78%, respectively. **Conclusions**: This study developed a pragmatic tool with three criteria for stratifying the GDM group not requiring insulin treatment, with successful validation for clinical use.

## 1. Introduction

Gestational diabetes (GDM) is characterised as a state of carbohydrate intolerance resulting in hyperglycaemia, and is associated with maternal adverse outcomes and neonatal morbidity, including shoulder dystocia, birth trauma, and macrosomia [1]. The prevalence of GDM has been rising steadily in the context of increasing maternal ages and obesity, lower thresholds for diagnosis, and more widespread testing [2,3]. The associated care is hugely burdensome for the obstetric care system, with annual costs of GDM care estimated at USD 5.6 billion in China [4]. Thus, stratifying care to those patients at most risk of disease complications and with the highest disease burden is a reasonable approach to optimise patient outcomes.

Maintaining blood glucose concentrations within the target range during pregnancies can reduce the risk of diabetes-related complications. Lifestyle interventions (diet and exercise) typically constitute the first management approach. If insufficiently effective, then pharmacological treatment is usually offered, typically with metformin or insulin [5]. Where both metformin and insulin are used, they are often introduced in a stepwise fashion such that those requiring insulin have more hyperglycaemia following diagnosis, consistent with having worse GDM-related outcomes to those not requiring additional insulin treatment [6,7]. However, insulin is not cost-effective compared to metformin alone [8]. More specialist expertise is required to ensure safe insulin administration, such as in teaching patients about proper injection techniques, insulin storage, adjusting insulin doses, and recognising and managing hypoglycaemia.

It is recognised that there are limited data on the use of personalised medicine in GDM care, and the use of a “one size fits all” approach does not reflect individual pregnancy outcomes for affected individuals [9]. Current recommendations [10] for the management of patients with GDM are applied irrespective of disease severity, and patients tend to be seen within specialist diabetes multidisciplinary clinics. To enable complex diabetes care to be focused on those patients at highest risk of complications, we proposed designing our care provision to stratify pathways into general or specialist obstetric care, with approximately half of our patients with GDM entering each pathway. For this to be practical, a pragmatic and simple stratification tool required development, specific to our patient population, which could be applied at the point of diagnosis and would have a low “false negative” to identify those patients requiring more complex diabetes care provision to be moved from an original lower-risk care pathway. Although models to stratify insulin requirement had been proposed in previous studies, few had developed simple, pragmatic stratification tools suitable for use in busy clinical settings [11,12,13]. Moreover, most existing stratification tools weigh false positive and false negative equally [14].

Therefore, we aimed to develop and validate a simple risk stratification tool that can be used at the time of GDM diagnosis in a busy clinical setting to identify patients at low risk and higher risk of insulin requirement.

## 2. Materials and Methods

### 2.1. Study Population and Design

The study population included (1) patients who were diagnosed with GDM by 75 g oral glucose tolerance test (OGTT), (2) patients whose treatment information was available in the electronic medical record, and (3) patients giving birth at St Thomas’ Hospital during 2019 (model development cohort) and from the second quarter of 2022 (end of the pandemic restrictions in the UK) to the first quarter of 2023 (internal validation cohort) or patients giving birth at Princess Royal University Hospital in 2022 (external validation cohort). GDM was diagnosed according to NICE criteria [10] if the woman had either a fasting plasma glucose concentration of 5.6 mmol/L or above or a 2 h plasma glucose concentration of 7.8 mmol/L or above [10].

Typical management of GDM at both hospitals was as follows. All patients diagnosed with GDM received lifestyle education including advice on eating a healthy, balanced diet, keeping to a healthy weight, staying active, and doing regular exercise. For patients who were unable to maintain adequate glycaemic control with lifestyle intervention only, metformin was recommended. If metformin was contraindicated, not tolerated, or unable to achieve satisfactory glycaemic control, insulin was used to control blood glucose concentrations. On occasion, insulin treatment was offered immediately, for example for some patients with fasting plasma glucose concentration above 7.0 mmol/L at diagnosis.

### 2.2. Ethical Approval

Patients’ data were collected as part of a clinical audit of patient care and a quality improvement project for the management of patients with gestational diabetes mellitus. The clinical audits were reviewed by, and registered with, the GSTT clinical governance team (7591 and 16167) and the Princess Royal University Hospital clinical governance team (OBS4/2022/PRUH). Members of the direct care team extracted individual patient data from electronic patient records (Badgernet), after which identifying patient details were removed; these data were used to support a quality improvement project to risk-stratify clinical care organisation. The NHS HRA (Health Research Authority) decision tool was used to confirm that the study did not require separate Research Ethics Committee approval (https://www.hra-decisiontools.org.uk/ethics/ assessed on 6 May 2025) as application of our findings to other clinical settings requires patient-specific cohort assessment; individualised clinical decisions about use of medication and provision of care are bespoken to each setting, rather than being generalisable from publication. As such, individual patient data are not available for sharing. The project was conducted according to the recommendations of the Healthcare Quality Improvement Partnership (https://www.hqip.org.uk/wp-content/uploads/2017/02/guide-to-managing-ethical-issues-in-quality-improvement-or-clinical-audit-projects.pdf accessed on 6 May 2025).

### 2.3. Candidate Predictors and Outcomes

We selected routinely obtained parameters at the times of GDM diagnosis as candidate variables for the prediction of insulin therapy, which included maternal age, parity, booking body mass index (BMI), the gestational age at GDM diagnosis, fasting blood glucose concentration, and 2 h blood glucose concentration from OGTT and HbA1c at diagnosis.

The primary outcome was defined as the requirement for insulin treatment. The secondary outcome was a composite of GDM-related perinatal complications, including obstetric anal sphincter injury (OASI), large-for-gestational-age neonate, shoulder dystocia, and neonatal unit admission. Any one of the four outcomes present was defined as confirming the presence of the composite outcome. OASI was defined as a third- or fourth-degree perineal tear. A large-for-gestational-age neonate was defined as birth weight above the 90th percentile adjusted for gestational age calculated using the Fetal Medicine Foundation algorithm [15].

### 2.4. Statistical Analysis

The continuous variables were reported as means ± SDs or medians and interquartile ranges (IQRs) according to whether these variables were distributed normally. The categorical variables were presented as numbers and percentages. The Shapiro–Wilk test was used to assess variable distribution for continuous data.

Univariate logistic regression was performed to determine the associations between candidate variables and the requirement for insulin. The candidate variables presenting statistical significance (*p* < 0.20) upon univariate analysis were then included in the initial multiple logistic regression model [16]. We used a stepwise forward selection method with the Akaike Information Criterion (AIC) to build the final predictive model with the remaining variables. To quantify the influence of each predictor on the risk of requiring insulin, the odds ratio (OR) and corresponding 95% confidence interval (95% CI) were estimated.

To evaluate the model performance, we calculated the area under the ROC curve (AUC) based on the final model in the development cohort. A nomogram was constructed based on the predictive model to present the effect of each variable on the probability of insulin requirement.

The stratification tool for insulin requirement was developed by dichotomising the selected variables. We estimated the sensitivity, specificity, positive predictive value (PPV), and negative predictive value (NPV) of the final risk stratification tool. We validated this stratification tool both in internal and external validation cohorts and reassessed its performance.

Multiple imputation was used to address the presence of missing data in the model development cohort. Five imputation sets were generated. The imputation model, with the assumption of data missing at random, included all baseline characteristics (except ethnicity) and all candidate predictors and outcomes. Model coefficients were averaged across the 5 repetitions using “MA.criteria” [17]. We used the 2.5 and 97.5 percentiles from 200 bootstrap samples as the limits of the 95% confidence intervals of model coefficients.

All statistical analyses were performed using R 4.0.3 (packages: rms (version 6.7.1), MASS (version 7.3.60), MAMI (version 0.9.13), mice (version 3.16.0), pROC (version 1.18.5)) [18]. Except for the univariate logistic regression analysis, we defined *p* < 0.20 as a criterion to select candidate variables into multiple logistic regression. *p* < 0.05 was considered as showing statistical significance in all other tests.

### 2.5. Sample Size Calculation

Sample size calculation was performed based upon the inclusion of seven variables in our initial prediction tool development. We assumed that 15% of our patients would require insulin treatment. Using the simple suggestion that 10 adverse events should be reported per variable tested, this would have required 467 patients to be included. However, this relatively simple approach has been contested [19]; therefore, we also used the pmsampsize code in Stata (Version 18.0), selecting an assumed c-statistic of 0.75. This suggested a final sample size for the development cohort of 572 patients. Our existing annual audit reported data from 617 patients for the development cohort year, which was therefore assumed to be of adequate power to determine this prediction tool.

Data from a second cohort of patients from the same hospital were used as an internal validation cohort as an alternative to data splitting or boot-strapping the original sample. The sample size calculation was performed retrospectively as it utilised only the three selected variables from the multivariable regression. Using the same assumptions as previously, the estimated sample size for this was 246; the risk of overfitting from the first model necessitated inclusion of a higher number of patients, and therefore, data were selected pragmatically from patients giving birth in the 9 months post pandemic restrictions to match care provision and clinical decision making as experienced by the development cohort.

## 3. Results

There were 617, 487, and 205 patients who fulfilled the inclusion criteria in the model development cohort, internal validation cohort, and external validation cohort, respectively.

### 3.1. Variable Selection and Model Development

The characteristics of patients with GDM requiring insulin and women not requiring insulin in the model development cohort are shown in Table 1. In the OGTT, 12.6% of the patients had high fasting blood glucose (≥5.6 mmol/L) only; 70.2% of the patients had high 2 h blood glucose (≥7.8 mmol/L) only; 17.2% of patients had both high fasting and 2 h blood glucose concentrations. A total of 81.7% of patients did not require insulin therapy. The median booking BMI in the insulin-treated group was 31.2 kg/m^2^ (IQR 26.7–35.5) compared to 26.1 kg/m^2^ (IQR 23.0–31.0) in the non-insulin-treated group. The median fasting blood glucose in the insulin-treated group was 5.8 mmol/L (IQR 5.2–6.5) compared to 5.0 mmol/L (IQR 4.6–5.5) in the non-insulin-treated group. Shoulder dystocia occurred in 3.5% of deliveries in the insulin-treated group compared to 0.8% in the non-insulin-treated group. Babies born large-for-gestational-age occurred for in 11.5% of the insulin-treated group and 7.0% in the non-insulin-treated group

**Figure 1:**
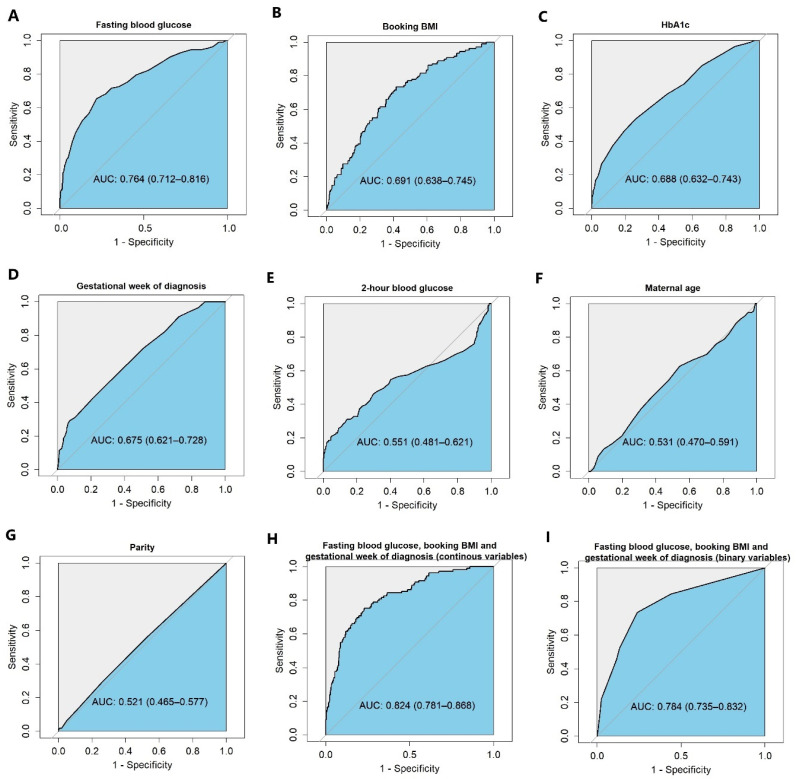
**The ROCs of logistic models predicting insulin requirement**. (**A**–**G**): ROC curves derived from univariate logistic regression model predicting insulin requirement. (**H**): The ROC curve of the multiple logistic regression model with three continuous variables (fasting blood glucose, booking BMI, and gestational week of GDM diagnosis). Model formula: Y = 0.974 × fasting blood glucose − 0.116 × gestational weeks at diagnosis + 0.047 × booking BMI − 5.258. (**I**): The ROC curve of the multiple logistic regression model with dichotomous variables (fasting blood glucose < 5.6 mmol/L or ≥5.6 mmol/L, booking BMI < 30 kg/m^2^ or ≥30 kg/m^2^ and gestational weeks at diagnosis ≥ 24 weeks or <24 weeks). ROC: receiver operating characteristic.

The associations between candidate predictors and insulin requirement were identified via univariate analysis separately (Table 2). The fasting blood glucose concentration, 2 h blood glucose concentration, HbA1c, booking BMI, and gestational weeks at the time of GDM diagnosis were significant predictors at the setting of *p* < 0.20. Fasting blood glucose had the greatest discriminative ability to predict whether patients would need insulin treatment (Figure 1). These variables were then included in the initial multiple logistic regression model. After applying a stepwise selection method with AIC criteria, HbA1c and 2 h blood glucose dropped out. In the final predictive model, patients with a higher fasting blood glucose concentration and higher booking BMI had an increased risk of requiring insulin therapy (OR 2.41, 95% CI 1.84–3.15 per mmol/L and OR 1.48, 95% CI 1.07–2.03 per kg/m^2^, respectively). A later gestational-weeks-at-diagnosis value had a lower risk of insulin therapy (OR 0.71, 95% CI 0.62–0.81 per week) (Table 2).

The missing data in the model development cohort are shown in Table S1. In the sensitivity analysis, we repeated the model selection process using five imputed datasets. The same three variables (fasting blood glucose, gestational weeks at GDM diagnosis, and booking BMI) were selected in the final predictive model (Table S2).

### 3.2. Developing a Simple Risk Stratification Tool to Stratify the Two Groups and the Performance of This Simple Risk Stratification Tool

The predictive performance of the model with continuous variables was shown by the area under the receiver operating characteristic curve (AUC ROC) to be 0.82 (95% CI 0.78 to 0.87) (Figure 1H). A nomogram was constructed based on the model with continuous variables to calculate the probability of insulin requirement for patients with GDM (Figure S1). A subgroup with a higher risk of insulin therapy was identified at the setting of 10% probability. This cutoff showed a sensitivity of 85.3%, specificity of 54.8%, PPV of 29.6%, and NPV of 94.4% (Table S3). A total of 284 (47%) patients were classified into the low-risk group, of whom 5.6% (n = 16/284) received insulin (Table S3).

In order to improve clinical utility, we dichotomised three variables separately (fasting blood glucose < 5.6 mmol/L or ≥5.6 mmol/L, booking BMI < 30 kg/m^2^ or ≥30 kg/m^2^, and GDM diagnosed before or after 24 weeks’ gestation) and built a predictive model with these categorical variables. These criteria were selected pragmatically from clinical experience and based on established diagnostic thresholds and the clinical delivery of care. These were very similar to the cutoffs chosen by the statistical method where Youden’s index is maximum (Figure S2). The performance of the model with binary variables was slightly compromised compared to the model with continuous variables, (ROC AUC 0.78, 95% CI 0.74 to 0.83) (Figure 1). We identified 291/598 (49%) of patients who did not have these risk factors for insulin requirement (fasting blood glucose < 5.6 mmol/L, booking BMI < 30 kg/m^2^, and gestational weeks at diagnosis ≥ 24 weeks) (Table 3) and defined this group as the low-risk group; those with any criterion increasing the likelihood of insulin requirement were defined as being in the high-risk group. This classification showed a specificity of 56.0%, sensitivity of 84.4%, PPV of 30.0%, and NPV of 94.2% (Table 3). The performance was similar to setting a 0.1 probability to cutoff values in the model with continuous variables (Table S3). Using this stratification, only 6% (17/291) of the low-risk group received insulin, OR 0.14 (95% CI 0.08–0.25). Compared to the high-risk group, they also had a lower risk of GDM-related composite adverse pregnancy outcomes (OR 0.53, 95% CI 0.34–0.82) (Table S4).

### 3.3. Internal and External Validation

The sensitivity (76.6%) and NPV (92.9%) of the stratification tool in the internal validation cohort were similar to those in the model development cohort (Table 4). By applying this tool, a total of 253 patients (52%) were classified as being in the low-risk group, of whom only 18 patients (7%) required insulin therapy, which was similar to the result from the model development cohort. The low-risk group identified by the simple tool had a lower risk of requiring insulin treatment (OR 0.23 95% CI 0.13–0.40), although no difference in the risk of GDM-related composite adverse birth outcomes was noted (OR 0.71, 95% CI 0.44–1.16) (Table S5).

The sensitivity and NPV of the stratification tool in the external validation cohort were 78.3% and 90.4%, respectively. The performance was similar to that in the model development cohort and internal validation cohort. The lower-risk group identified by the simple tool in the external cohort had a similarly lower risk of requiring insulin therapy (OR 0.19, 95% CI 0.09–0.41) but not a lower risk of experiencing any of the composite adverse outcomes (OR 0.60, 95% CI 0.27–1.32), consistent with the internal validation cohort.

## 4. Discussion

We developed a pragmatic tool to identify a low-risk group of patients with GDM not requiring insulin treatment; this tool was successfully validated with internal and external patient cohorts. Using three stratification criteria at diagnosis (booking BMI < 30 kg/m^2^, fasting blood glucose < 5.6 mmol/L, and gestational weeks at diagnosis ≥ 24 weeks), almost half of the patients with GDM were at low risk of requiring insulin, enabling the stratification of patients’ care pathways.

One strength of this study was that our simple risk stratification tool used predictors present at GDM diagnosis, and we were reassured that the tool performed similarly for patients with GDM after the COVID pandemic (during which antenatal care modifications occurred). External validation from a district general hospital, but also within a London borough, similarly demonstrated the utility of our simple model. A further strength of this study was the mixed ethnicity in our populations, which made this tool more likely to be applicable to a broader range of settings.

There were several limitations of our study. Our tool was developed for GDM diagnosed by NICE criteria; it is unclear how well this tool works for populations diagnosed using different criteria. Another limitation was the retrospective study design based on the data existing in the electronic patient records.

Multiple factors have previously been identified as predictors for insulin requirement at different stages of pregnancy [11,12,13,20]. Aiming to develop a pragmatic model to be applied at the time of GDM diagnosis, we selected the readily accessible maternal characteristics and biochemical variables as candidate predictors. It is striking that our “real world” data identified variables predictive of subsequent insulin requirement similar to those identified in a recent systematic review of personalised medicine approaches in gestational diabetes approaches [21]; we support the suggestion of these authors that future research should harness more complex “omic” technology to further advance the application of personalised medicine within GDM management.

The three variables included in our final models had all been identified as risk factors for insulin requirement in previous studies [13]. Despite use in different patient population and with different GDM diagnostic criteria, that fasting blood glucose was the strongest predictor of insulin requirement was consistent with previous work [11,22,23,24]; this was also tenable in early-onset GDM [14]. Whether the inclusion of other markers of hyperglycemia in predictive models is of benefit differs between studies: HbA1c and post-carbohydrate glucose measurements in the OGTT were not additionally relevant for us or for Akinci’s model [25]. However, Barnes [26] reported that HbA1c retained significance in the multivariable regression model, and others have found both HbA1c and 2 h glucose are independent predictors of insulin requirement after adjustment for fasting blood glucose [20,27]. These inconsistencies suggest the value of developing tools appropriate to the target population.

Although several studies had developed a risk prediction model for the necessity of insulin therapy, none had specially aimed to identify a lower-risk-of-GDM group. A common stratification method was setting a probability cutoff after predictive models were established; some studies chose to set a cutoff of 50% probability of insulin requirement, implying that false positive and false negative classifications are equally important; however, Greenland [28] argues that missing a patient with the event is usually more important than the incorrect classification of a patient without the outcome. Sapienza [29] stratified two groups by setting a cutoff value of 30% probability of insulin requirement: 177 patients had less than 0.3 probability of requiring insulin; however, 42.4% and 44.6% of 177 patients received insulin treatment based on two separate models, which was not adequate for our clinical need. Accordingly, we set a 0.9 probability of not requiring insulin as a cutoff to identify the “low-risk” patients. This cutoff classified almost half (46%) of our patients into the low-risk group, of whom only 5.9% received insulin.

Many predictive models in GDM care with good AUCs are hard to translate into clinical use as they include variables not routinely measured, such as sex hormone binding globulin (SHBG) [30]. Some studies have reported complex equations including numerous predictors, which make their models unfeasible to apply with current technology in the busy clinical setting, including those models developed using supervised machine learning algorithms [12]. Instead of setting a cutoff probability, some studies have categorised the variables for easier clinical application. Barnes [26] dichotomised seven predictors of insulin requirement although they did not define low- and high-risk groups. Eleftheriades [22] introduced a classification tree method, by which eight groups of patients with GDM were separated by risk of insulin requirement; 67% of patients formed the lowest-risk group, with 8% probability of insulin requirement; however, the performance measures (sensitivity, specificity, PPV, and NPV) of these criteria were not reported. Koefoed [31] developed a point score system, and 642 (58.2%) patients were categorised into a “low-risk group” with a cutoff score < 3; however, of these, 77 (27.3%) of these still required insulin treatment. The tool that we proposed classified almost half (49%) of our patients into the low-risk group, of whom only 6% received insulin. In order to avoid many false negatives (patients requiring insulin who are misclassified into the low-risk group), the tool has a relatively low specificity (56%) with 44% of patients who do not require insulin being allocated to the high-risk group (false positive). For the organisation of our clinical service, we believe that this trade-off is justified; the unique nature of this simple predictive tool is that it differs from many others, which aim to maximize specificity at the expense of sensitivity.

Given that smoking has been associated with increased insulin resistance and higher rates of insulin requirement in GDM [32], for populations with higher background rates of pre-pregnancy smoking, this could be included in future iterations of this predictive model; locally, the South East London patient group has low, and falling, rates of smoking in pregnancy, and therefore, the inclusion of this as another variable would be unlikely to have additional benefit.

## 5. Conclusions

In summary, we have demonstrated the use of routinely collected data to enable the development of a risk stratification tool to identify patients with GDM at low risk of needing insulin treatment; the validity of the tool can be applied to a similar population within the region. Future work will assess the clinical impact of adoption of this tool within the clinical setting.

## 6. Patients

The study population included the following: (1) patients who were diagnosed with GDM by 75 g oral glucose tolerance tests (OGTTs), (2) patients whose treatment information was available in the electronic medical record, and (3) patients giving birth at St Thomas’ Hospital during 2019 (model development cohort) and from the second quarter of 2022 (end of the pandemic restrictions in the UK) to the first quarter of 2023 (internal vali-dation cohort) or patients giving birth at Princess Royal University Hospital in 2022 (external validation cohort). Patients’ details were included as part of a clinical audit of patient care and a quality improvement project for the management of patients with gestational diabetes mellitus. Following extraction from electronic patient records (Badgernet), identifying patient details were removed by pseudoanonymising data. The audits were registered with the GSTT clinical governance team (7591 and 16167) and the Princess Royal University Hospital clinical governance team (OBS4/2022/PRUH).

## Figures and Tables

**Table 1:** Comparison of characteristics between insulin treatment group and no-insulin-treatment group in model development cohort.

	No Insulin Treatment (n = 504)	Insulin Treatment (n = 113)
**Maternal characteristics**		
Maternal age (years), median (IQR)	34 (31, 38)	35 (31, 38)
Ethnicity (n, %)		
Asian	110 (21.8)	21 (18.6)
Black	120 (23.8)	21 (18.6)
Mixed	13 (2.6)	7 (6.2)
White	138 (27.4)	38 (33.6)
Unknown	83 (16.5)	19 (16.8)
None of the above	40 (7.9)	7 (6.2)
Parity (n, %)		
Primipara	234 (46.4)	49 (43.4)
Multipara	270 (53.6)	64 (56.6)
Booking BMI (kg/m^2^), median (IQR)	26.1 (23.0, 31.0)	31.2 (26.7, 35.5)
Fasting blood glucose (mmol/L), median (IQR)	5.0 (4.6, 5.5)	5.8 (5.2, 6.5)
2 h blood glucose (mmol/L), median (IQR)	8.4 (8.0, 9.3)	8.8 (7.8, 10.6)
Gestational week of GDM diagnosis (weeks),median (IQR)	26 (26, 29)	26 (22, 27)
HbA1c (mmol/mol), median (IQR)	46.4 (44.7, 49.0)	49.0 (45.6, 52.5)
**Pregnancy outcomes**		
Preterm birth (n, %)		
No	455 (90.6)	105 (92.9)
Yes	47 (9.4)	8 (7.1)
Mode of birth (n, %)		
Vaginal birth	281 (56.0)	51 (45.1)
Caesarean birth	221 (44.0)	62 (54.9)
Shoulder dystocia (n, %)		
No	498 (99.2)	109 (96.5)
Yes	4 (0.8)	4 (3.5)
Neonatal unit admission (n, %)		
No	464 (92.4)	103 (91.2)
Yes	38 (7.6)	10 (8.8)
Large for gestational age (n, %)		
No	467 (93.0)	100 (88.5)
Yes	35 (7.0)	13 (11.5)
Obstetric anal sphincter injury (n, %)		
No	491 (97.8)	112 (99.1)
Yes	11 (2.2)	1 (0.9)
Apgar < 7 at 5 min, (n, %)		
No	483 (98.4)	109 (97.3)
Yes	8 (1.6)	3 (2.7)
Foetal birth outcome (n, %)		
Live birth	498 (99.2)	112 (99.1)
Stillbirth	3 (0.6)	1 (0.9)
Neonatal death	1 (0.2)	0 (0.0)

IQR, inter-quartile range.

**Table 2:** Univariate, multivariate, and model selection results of logistic regressions for predicting insulin treatment.

	Univariate *		Multivariate †		Model Selection †	
	OR (95% CI)	*p*	OR (95% CI)	*p*	OR (95% CI)	*p*
Maternal age (years)	1.10 (0.84–1.44)	0.49	–	–	–	–
Parity (n%)						
Primipara	Ref					
Multipara	0.88 (0.59–1.33)	0.55	–	–	–	–
Booking BMI (kg/m^2^)	2.35 (1.79–3.09)	<0.001	1.55 (1.11–2.17)	0.011	1.48 (1.07–2.03)	0.017
Fasting blood glucose (mmol/L)	2.64 (2.06–3.38)	<0.001	2.35 (1.67–3.30)	<0.001	2.41 (1.84–3.15)	<0.001
2 h blood glucose (mmol/L)	1.37 (1.19–1.56)	<0.001	1.10 (0.91–1.34)	0.32	–	–
Gestation of GDM diagnosis (weeks)	0.66 (0.58–0.74)	<0.001	0.70 (0.61–0.81)	<0.001	0.71 (0.62–0.81)	<0.001
HbA1c (mmol/mol)	1.95 (1.57–2.42)	<0.001	0.94 (0.68–1.30)	0.71	–	–

* Univariate analyses were performed with the unimputed data. † Analyses were performed in the 592 patients with all the 5 variables available (booking BMI, fasting blood glucose, 2 h blood glucose, HbA1c, and gestational weeks at GDM diagnosis). OR: odds ratio; CI: confidence interval.

**Table 3:** Sensitivity, specificity, positive predictive value, and negative predictive value data of the stratification tool in model development cohort.

	Actual Treatment		
Tool Predicted Insulin Need	Insulin	No Insulin	Total
High-risk group	92	215	307
Low-risk group	17	274	291
Total	109	489	

Sensitivity = 84.4%. Specificity = 56.0%. Positive predictive value (PPV) = 30.0%. Negative predictive value (NPV) = 94.2%. These results were analysed from 598 women with all the three variables available (fasting blood glucose, BMI, and gestational weeks at diagnosis).

**Table 4:** The performance of stratification tool in internal and external validation cohorts.

	Internal Validation			External Validation		
	Actual Treatment			Actual Treatment		
Tool Predicted Insulin Need	Insulin	No Insulin	Total	Insulin	No Insulin	Total
High-risk group	59	174	233	36	65	101
Low-risk group	18	235	253	10	94	104
Total	77	409		46	159	
Sensitivity = 76.6% Specificity = 57.5% Positive predictive value (PPV) = 25.3% Negative predictive value (NPV) = 92.9%				Sensitivity = 78.3% Specificity = 59.1% Positive predictive value (PPV) = 35.6% Negative predictive value (NPV) = 90.4%		

Internal validation was analysed among 486 women with all the three variables available (fasting blood glucose, BMI, and gestational weeks at GDM diagnosis).

## Data Availability

Data were collected as part of registered clinical audits at both St Thomas’ Hospital and Princess Royal University Hospital (GSTT audit IDs: 7591 and 16167, PRUH audit ID: OBS4/2022/PRUH). Individual participant data would not be available for data sharing, although summary data would be available at reasonable request to the corresponding author.

## Supplementary Materials

The following supporting information can be downloaded at: https://www.mdpi.com/article/10.3390/jpm15060223/s1, Figure S1: Nomogram representing the results of the logistic regression model for the prediction of insulin requirement; Figure S2: The cutoff of each variable based on where the Youden’s index was maximum; Table S1: Characteristics of data used for model development cohort and proportion with missing data; Table S2: Multiple logistic regression results of predicting insulin requirement after data imputation; Table S3: The performance of classification based on the cutoff of 0.1 probability of insulin treatment; Table S4: The risk of insulin treatment and GDM-related composite adverse outcome in model development cohort; Table S5: The risk of insulin treatment and GDM-related adverse outcome in validation cohorts.

## Abbreviations

The following abbreviations have been used in this manuscript:

GDM	Gestational diabetes mellitus
OR	Odds ratio
NPV	Negative predictive value
OGTT	Oral glucose tolerance test
OASI	Obstetric anal sphincter injury
IQR	Interquartile range
AIC	Akaike Information Criterion
AUC	Area under the ROC curve
PPV	Positive predictive value
CI	Confidence interval
BMI	Body mass index
