# Supplementary material for: Developing a Risk Stratification Tool to Predict Patients with Gestational Diabetes Mellitus at Risk of Insulin Treatment: A Cohort Study
Source: J Pers Med. 2025 May 30;15(6):223. doi: 10.3390/jpm15060223 (PMC12194323; doi:10.3390/jpm15060223)
Supplement: Supplementary file 1 [file jpm-15-00223-s001.zip › Suplementary_file.pdf]

Supplementary Tables

Table S1. Characteristics of data used for model development cohort and proportion with missing data

	**Development cohort (*n* = 617)**	
	**Median [IQR] or *n* (%)**	**Number with missing data, *n* (%)**
Maternal characteristics		
Maternal age (years)	34 [31, 38]	2 (0.3)
Ethnicity (n%)		102 (16.5)
Black	141 (27.4)	
Asian	131 (25.4)	
White	176 (34.2)	
Mixed	20 (3.9)	
Other	47 (9.1)	
Parity(n%)		0 (0.0)
Primipara	283 (45.9)	
Multipara	334 (54.1)	
Booking BMI (kg/m^2)	27.0 [23.5, 31.8]	19 (3.1)
Fasting blood glucose (mmol/l)	5.1 [4.7, 5.6]	0 (0.0)
2-hour blood glucose (mmol/l)	8.5 [8.0, 9.4]	0 (0.0)
Gestational week of GDM diagnosis, median [IQR]	26 [25, 28]	0 (0.0)
HbA1c (mmol/mol), median [IQR]	46.4[44.7, 49.0]	6 (1.0)
Pregnancy outcomes		
Preterm birth (n, %)		2 (0.3)
No	560 (91.1)	
Yes	55 (8.9)	
Delivery method (n, %)		2 (0.3)
Vaginal delivery	332 (54.0)	
Caesarean delivery	283 (46.0)	
Shoulder dystocia (n, %)		2 (0.3)
No	607 (98.7)	

Yes	8 (1.3)	
Neonatal unit admission (n, %)		2 (0.3)
No	567 (92.2)	
Yes	48 (7.8)	
Large for gestational age (n, %)		2 (0.3)
No	567 (92.2)	
Yes	48 (7.8)	
OASI (n, %)		2 (0.3)
No	603 (98.0)	
Yes	12 (2.0)	
Apgar<7 score, (n, %)		14 (2.3)
No	592 (98.2)	
Yes	11 (1.8)	
Fetal birth outcome (n, %)		2 (0.3)
Live birth	610 (99.2)	
Stillbirth	4 (0.7)	
Neonatal death	1 (0.2)	

Table S2. Multiple logistic regression results of predicting insulin requirement after data imputation

	**Full Model**	**Model Selection***
Variables	**OR (95%CI)**	**OR (95%CI)**
Booking BMI (kg/m^2)	1.05 (1.00-1.10)	1.05 (1.01-1.09)
Fasting blood glucose (mmol/l)	2.42 (1.76-3.33)	2.48 (1.87-3.29)
2-hour blood glucose (mmol/l)	1.03 (0.93-1.14)	------
Gestational week of GDM diagnosis	0.89 (0.85-0.94)	0.89 (0.86-0.94)
HbA1c (mmol/mol)	1.00 (0.96-1.04)	------

* Using AIC and model averaging

OR, odds ratio; CI, confidence interval.

Table S3. The performance of classification based on the cutoff of 0.1 probability of insulin treatment

	**Actual treatment**

Model’s predicted insulin need	Inulin	No insulin	Total
High risk group, P (insulin) >0.1	93	221	314
Low risk group, P (insulin) ≤0.1	16	268	284
Total	109	489	

Sensitivity=85.3%

Specificity=54.8%

Positive predictive value (PPV) =29.6%

Negative predictive value (NPV)= 94.4%

598 women that all the three predictors are available (fasting blood glucose, booking BMI and gestational week at diagnosis) from model development cohort were analysed in Table S3.

Table S4. The risk of insulin treatment and GDM-related composite adverse outcome in model development cohort

Groups	**Insulin treatment OR (95%CI)**	**Composite outcome OR (95%CI)**
High risk criteria (n=307)	Reference	Reference
Low risk criteria (n=291)	0.14(0.08-0.25)*	0.53(0.34-0.82)*

*p<0.05, OR: odds ratio, CI: confidence interval

598 women with all the three variables available (fasting blood glucose, booking BMI and gestational week at diagnosis) from model development cohort were analysed in table 4.

Table S5. The risk of insulin treatment and GDM-related adverse outcome in validation cohorts

	**Groups**	**Insulin treatment OR (95%CI)**	**Composite outcome OR (95%CI)**
Internal validation cohort	High risk criteria (n=233)	Reference	Reference
	Low risk criteria (n=253)	0.23 (0.13-0.40)*	0.71 (0.44-1.16)
External validation cohort	High risk criteria (n=101)	Reference	Reference
	Low risk criteria (n=104)	0.19 (0.09-0.41)*	0.60 (0.27-1.32)

*p<0.05, OR: odds ratio, CI: confidence interval.

Internal validation was analysed among 486 women with all the three variables available (fasting blood glucose, BMI and gestational week at GDM diagnosis).

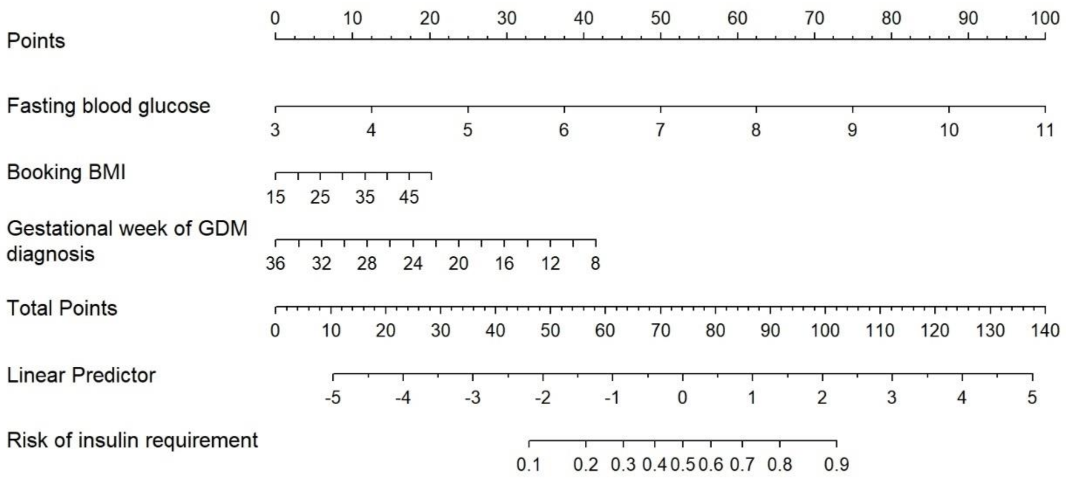

Figure S1. Nomogram representing the results of the logistic regression model for the prediction of insulin requirement

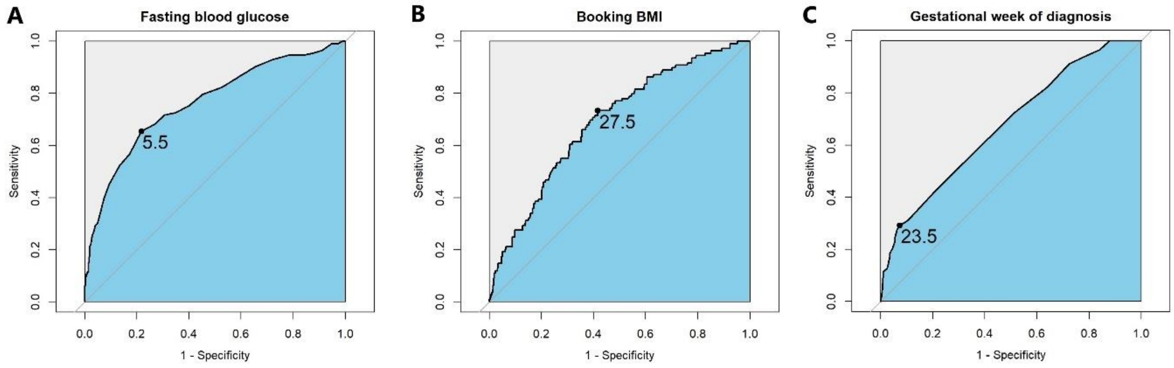

Figure S2. The cutoff of each variable based on where the Youden’s index is maximum
